# Using Arterial Pulse and Laser Doppler Analyses to Discriminate between the Cardiovascular Effects of Different Running Levels

**DOI:** 10.3390/s23083855

**Published:** 2023-04-10

**Authors:** Yi-Jia Lin, Chia-Chien Lee, Tzu-Wei Huang, Wei-Chun Hsu, Li-Wei Wu, Chen-Chun Lin, Hsin Hsiu

**Affiliations:** 1 Graduate Institute of Biomedical Engineering, National Taiwan University of Science and Technology, Taipei 106, Taiwan; 2 Division of Family Medicine, Department of Family and Community Medicine, Tri-Service General Hospital, School of Medicine, National Defense Medical Center, Taipei 114, Taiwan; 3 Health Management Center, Department of Family and Community Medicine, Tri-Service General Hospital, National Defense Medical Center, Taipei 114, Taiwan; 4 College of Applied Science, National Taiwan University of Science and Technology, Taipei 106, Taiwan

**Keywords:** running, pulse, laser-doppler, blood flow, spectral analysis, cardiovascular variability

## Abstract

Background and Aims: Running can induce advantageous cardiovascular effects such as improved arterial stiffness and blood-supply perfusion. However, the differences between the vascular and blood-flow perfusion conditions under different levels of endurance-running performance remains unclear. The present study aimed to assess the vascular and blood-flow perfusion conditions among 3 groups (44 male volunteers) according to the time taken to run 3 km: Level 1, Level 2, and Level 3. Methods: The radial blood pressure waveform (BPW), finger photoplethygraphy (PPG), and skin-surface laser-Doppler flowmetry (LDF) signals of the subjects were measured. Frequency-domain analysis was applied to BPW and PPG signals; time- and frequency-domain analyses were applied to LDF signals. Results: Pulse waveform and LDF indices differed significantly among the three groups. These could be used to evaluate the advantageous cardiovascular effects provided by long-term endurance-running training, such as vessel relaxation (pulse waveform indices), improvement in blood supply perfusion (LDF indices), and changes in cardiovascular regulation activities (pulse and LDF variability indices). Using the relative changes in pulse-effect indices, we achieved almost perfect discrimination between Level 3 and Level 2 (AUC = 0.878). Furthermore, the present pulse waveform analysis could also be used to discriminate between the Level-1 and Level-2 groups. Conclusions: The present findings contribute to the development of a noninvasive, easy-to-use, and objective evaluation technique for the cardiovascular benefits of prolonged endurance-running training.

## 1. Introduction

It is widely accepted that running is associated with reduced cardiovascular risks, even for short times such as 5–10 min/day and at slow speeds of <6 miles/h [1,2]. Trained marathon runners have been found to measure up to the increased cardiovascular demands better than untrained subjects [3]. Furthermore, compared with powerlifters, the cardiac performance of long-distance runners has been found to be associated with improved vascular function (e.g., reduced arterial stiffness and oxidative stress and increased endothelium-dependent dilation capacity) induced by aerobic exercise training [4].

Important advantageous cardiovascular effects provided by exercise include the following [5]: lowering heart rate (HR) [5,6,7], improving VO_2_max, increasing cardiac output, favorable responses to acute inflammatory challenges [5], and better structural myocardium adaptations [8]. The objective evaluation of these positive effects may help to determine the physiological advantages, including the reduced cardiovascular burden, such as reducing the risk of cardiovascular diseases (CVDs) [1,5]. Vessel relaxation, which causes conditions such as stimulated NO production, reduced blood pressure (BP), increased insulin sensitivity, and a more favorable plasma lipoprotein profile [5], has also been reported. Decreased pulse wave velocity (PWV) [7,9], decreased arterial stiffness [4,9,10], and an improved elastin-to-collagen ratio within the extracellular matrices of central arteries [9] have also been noted, while improvement in blood supply perfusion causes increased blood flow perfusion [5] and capillary density of skeletal muscles in distance runners [2].

Despite the benefits, studies have also found that prolonged endurance running can induce adverse changes in the elastic properties of vessels. The underlying mechanism may be related to mechanical fatigue caused by the repeated and excessive stress imposed on the elastic elements of the vessel wall. Changes in the adaptive responses of the cardiovascular system to the demand in trained marathon runners may induce increased HR and peripheral vasodilation of muscular arteries and arterioles [3]. The systemic inflammation induced by intense exercise training might increase arterial stiffness [2,3]. Another possible mechanism for arterial stiffening is the increased sympathetic vascular tone after short-term training [2]. Changes in cardiovascular regulation activities have also been found to increase time-domain heart rate variability (HRV) parameters in athletes who underwent long-term training. Decreased HRV can also be used as a marker of overtraining and exhaustion [6].

Since arterial pulses are transmitted along arteries, changes in their elastic properties and hence in the condition of blood-flow perfusion can be evaluated using pulse waveform measurements and analyses. Furthermore, arterial pulse waveforms can be measured easily using noninvasive methods such as the radial blood pressure waveform (BPW) and photoplethygraphy (PPG) signals. In previous studies, changes in the elastic properties of the vascular system induced by aging or cardiovascular diseases (CVDs) have been investigated using time-domain indices of pulse waveforms [11,12]. Since arterial stiffness quantified by the carotid-femoral PWV has been considered a valid vascular biomarker, it is acceptable for use as an optimized individual cardiovascular risk stratification following guideline recommendations [13,14,15,16]. Another category of waveform analysis is frequency-domain analysis. The effects induced on pulse waveforms have also been studied to determine the possible vascular effects on subjects with vascular aging, frozen shoulder, sarcopenia, and Alzheimer’s disease, as well as those who received COVID-19 vaccination [17,18,19,20,21,22].

Laser Doppler flowmetry (LDF) is a noninvasive method that utilizes the optical signal reflected from the tissue to evaluate the blood-flow perfusion. Various analysis types have also been applied to LDF waveforms. Time-domain analysis of beat-to-beat LDF waveforms can aid the evaluation of perfusion resistance and efficiency through arteriolar openings [22], while frequency-domain analysis can be used to determine the activities of several regulatory mechanisms [23]. LDF measurements and analyses have been used to monitor the responses to blood-flow perfusion induced by exercise, such as running and fitness [24,25,26].

The ways in which prolonged running training and the endurance-running level affect vascular function is a matter of ongoing debate [27]. The aim of the present study was therefore to combine noninvasive arterial pulse and LDF measurements and analyses to assess the vascular and blood-flow perfusion conditions; the pulse and LDF indices were compared among three groups: Level 1, Level 2, and Level 3. Physiological benefits have been noted in running for Level 2 [4,7,8], and it is possible that the Level-1 group have better running performance and blood flow perfusion. This comparison may help in the development of indices to facilitate a noninvasive, easy-to-use, and objective technique for evaluating the physiological benefits of exercise. It was hypothesized that endurance running induces changes in hemodynamics by reflecting the differences in hemodynamic condition depending on the 3-km running performance of individual runners. We expected hemodynamic and inflammatory processes to be the underlying mediators for these changes.

## 2. Materials and Methods

### 2.1. Participants

Measurements were performed on 44 male volunteers, whose characteristics are detailed in Table 1. The participants were divided into the following three groups: participants in the Level-1 group could run 3 km within 12 min, those at Level 2 could run 3 km in 12–15 min, and those at Level 3 required longer than 15 min to run 3 km.

We recruited only male runners because running mechanics have been found to differ between the sexes [28]. The other inclusion criteria were (1) aged 18–40 years; (2) being able to run a minimum of 15 km per week as regular running, which has been reported to minimize injury risk [29]; (3) being able to run 5 km in less than 23 min [30]. The exclusion criteria were (1) a history of arrhythmia or heart disease; (2) a history of lower limb surgery; or (3) having experienced neuromusculoskeletal injury within the previous 6 months.

### 2.2. Outdoor 3-km Testing

Participants wore shoes while running 3 km on a 200-m outdoor running track. Participants completed a 10-min warm-up before the test, which included static stretching, slow running, and dynamic stretching. The participants rested for 10–15 min after the warm-up. An accelerometer sensor was attached to the subjects, and the Rating of Perceived Exertion (RPE) on a scale from 1 to 10 could not be higher than 3 before the test was started.Furthermore, an infrared time gate that we developed was used to record the completion time of each lap. 

### 2.3. Measurement

**Figure 1:**
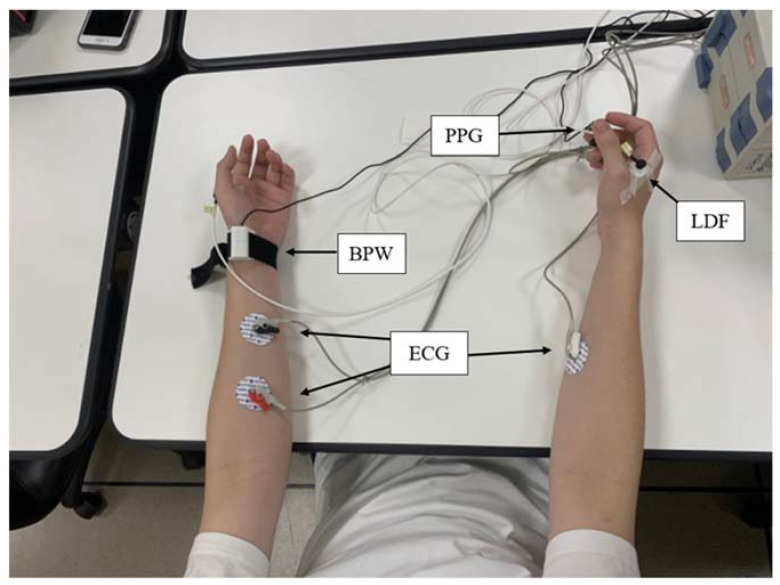
Experimental setup.

**Figure 2:**
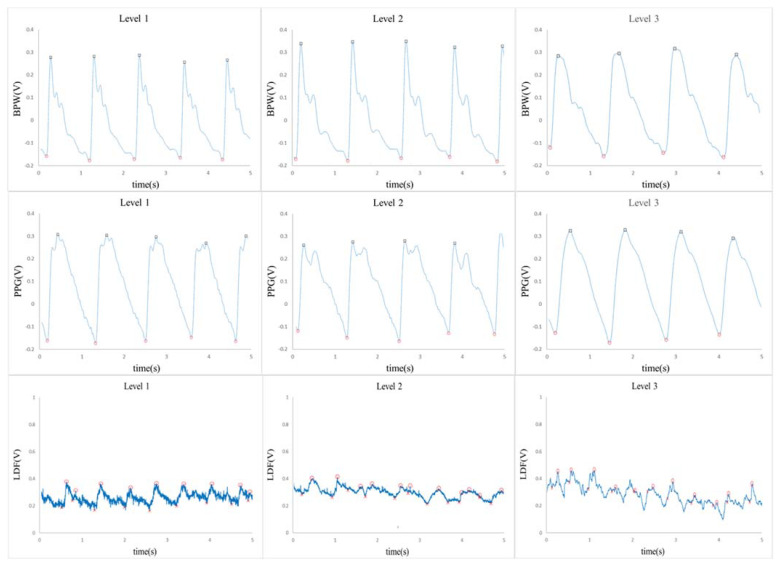
Typical time-domain waveforms.

Details of the present experimental setup and the signal processing methods are available elsewhere [20,31] and in the Supplemental Materials. Radial BPW, finger PPG and skin-surface LDF signals of the subjects were measured noninvasively (experimental setup is shown in Figure 1; typical waveforms are shown in Figure 2). Written informed consent was obtained from each study participant (approved by the Research Ethics Committee of Tri-Service General Hospital; TSGHIRB 2-108-05-161), and all experiments were performed in accordance with relevant guidelines and regulations.

Before the measurements, the subjects were relaxed and rested for at least 10 min. The environmental temperature was within 23–25 °C during the entire measuring period. For each experiment, the subjects were sitting on a chair, and 1-min BPW and PPG signals were measured noninvasively, followed by a 20-min LDF measurement. The BPW signal was acquired by a pressure transducer (KFG-2-120-D1-11, Kyowa) held onto the skin surface above the radial artery, 2 cm from the left wrist. The PPG signals from a 940-nm-wavelength infrared LED penetrating the middle finger tissue were acquired by photodiodes. A Moor VMS-LDF (VP1 probe; MBF3, Moor Instruments, Devon, UK) was used to measure the skin-surface temperature and the MBF flux between the thumb and the index finger on the back of the hand. The signals were connected to a self-made current-to-voltage converter circuit, and then connected to an analog-to-digital converter card (PCI-9111DG, Adlink Technology, Taipei, Taiwan) operating at a sampling rate of 1024 Hz.

### 2.4. Analysis

For BPW and PPG signals, frequency-domain analysis was performed to derive the following 40 harmonic indices for *n* = 1–10: amplitude proportion (*C_n_*), coefficient of variation in *C_n_* (*CV_n_*), phase angle (*P_n_*), and standard deviation of *P_n_* (*P_n_*_*SD*) [17,18,19,20,21]. Pulse indices were further used to determine the discrimination ability by utilizing the following self-developed analyses of the relative changes in the pulse effect indices:

- Choose the indices with significant or prominent differences relative to the Level-3 group.
- Prepare to calculate the pulse effect indices: divide the values by the average value of the Level-3 group for *C*_2_, *C*_4_, *C*_5_, *C*_6_, *C*_7_, *P*_2_, *P*_3_, *P*_4_, *P*_5_, *P*_6_…. and denote them as *C*_2′_, *C*_4′_, ….
- Define the amplitude effect index as (*C*_4′_ × *C*_5′_ × *C*_6′_ × *C*_7′_/*C*_2′_).
- Define the angle effect index as (*P*_2′_ × *P*_3′_ × *P*_4′_ × *P*_5′_ × *P*_6′_).
- Plot the relative changes in the pulse effect indices. The relative change is based on the concept of Z-score standardization. With the amplitude effect index as an example, the relative change was defined as ((one amplitude effect index) − (average of all amplitude effect indices of the group))/(average of all amplitude effect indices of the group).

## 3. Results

**Figure 3:**
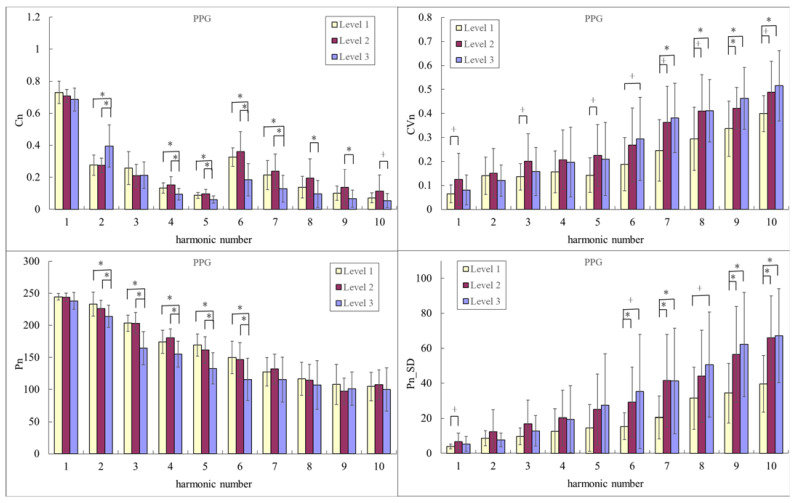
Comparisons of PPG harmonic indices between Level 3, Level 2, and Level 1: *C_n_*, *CV_n_*, *P_n_*, and *P_n_*_*SD*. Data are mean and SD values. *C*_5_–*C*_10_ values have been multiplied by 5 to make the differences clearer. “*” indicates *p* < 0.05; “+” indicates 0.05 < *p* < 0.1. For *C_n_* values, *C*_2_ was significantly smaller in Level 2 and Level 1 than in Level 3, *C*_4_–*C*_7_ were significantly larger in Level 2 and Level 1 than in Level 3, and *C*_8_ and *C*_9_ were significantly larger in Level 2 than in Level 3. For *P_n_* values, *P*_2_–*P*_6_ were significantly larger in Level 2 and Level 1 than in Level 3. Regarding *CV_n_* values, *CV*_1_, *CV*_3_, and *CV*_5_ appeared to be smaller in Level 1 than in Level 2, and *C*_7_–*C*_10_ were significantly smaller or appeared to be smaller in Level 1 than in Level 2 and Level 3. Regarding *P_n__SD* values, *P*_6_*_SD*, *P*_7_*_SD*, *P*_9_*_SD*, and *P*_10_*_SD* were significantly smaller or appeared to be smaller in Level 1 than in Level 2 and Level 3.

Figure 3 compares PPG harmonic indices among the Level-3, Level-2, and Level-1 groups. Regarding *C_n_* values, *C*_2_ was significantly smaller, and several higher-frequency components (e.g., *C*_4_–*C*_9_) were significantly larger in Level 2 and Level 1 than in Level 3. Regarding *P_n_* values, *P*_2_–*P*_6_ were significantly larger in Level 2 and Level 1 than in Level 3. Several of the PPG variability indices were significantly smaller or appeared to be smaller in Level 1 than in Level 2 and Level 3 (e.g., *CV*_1_, *CV*_3_, *CV*_5_–*CV*_10_, *P*_1_*_SD*, and *P*_6_*_SD*–*P*_10_*_SD*).

**Figure 4:**
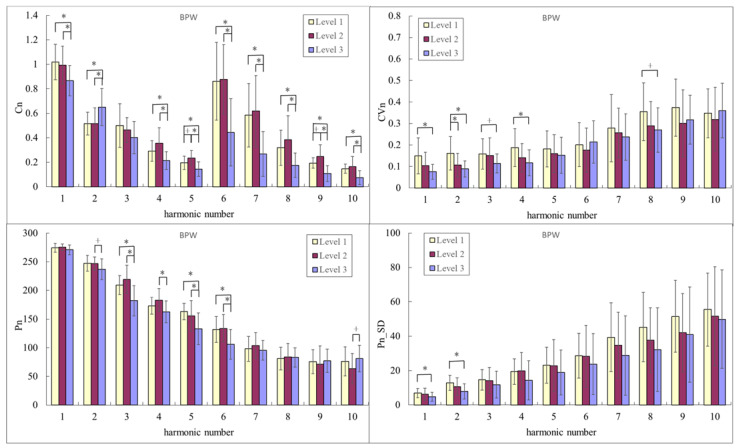
Comparisons of BPW harmonic indices between Level 3, Level 2, and Level 1: *C_n_*, *CV_n_*, *P_n_*, and *P_n_*_*SD*. Data are mean and SD values. *C*_5_–*C*_10_ values have been multiplied by 5 to make the differences clearer. “*” indicates *p* < 0.05; “+” indicates 0.05 < *p* < 0.1. For *C_n_* values, *C*_2_ was significantly smaller in Level 2 and Level 1 than in Level 3, and *C*_1_ and *C*_4_–*C*_10_ were significantly larger in Level 2 and Level 1 than in Level 3. For *P_n_* values, most of *P*_3_–*P*_6_ were significantly larger in Level 2 and Level 1 than in Level 3. For *CV_n_* values, *CV*_1_–*CV*_4_ and *CV*_8_ were significantly larger or appeared to be larger in Level 1 than in Level 3. For *P_n__SD* values, *P*_1_*_SD* and *P*_2_*_SD* were significantly larger in Level 1 than in Level 3.

Figure 4 compares BPW harmonic indices among the Level-3, Level-2, and Level-1 groups. For *C_n_* values, *C*_2_ was significantly smaller while *C*_1_ and *C*_4_–*C*_10_ were significantly larger in Level 2 and Level 1 than in Level 3. For *P_n_* values, *P*_3_–*P*_6_ were significantly larger in Level 2 and Level 1 than in Level 3. Several of the BPW variability indices were significantly larger in Level 1 than in Level 3 (e.g., *CV*_1*,*_ *CV*_2_, *P*_1_*_SD*, and *P*_2_*_SD*).

**Figure 5:**
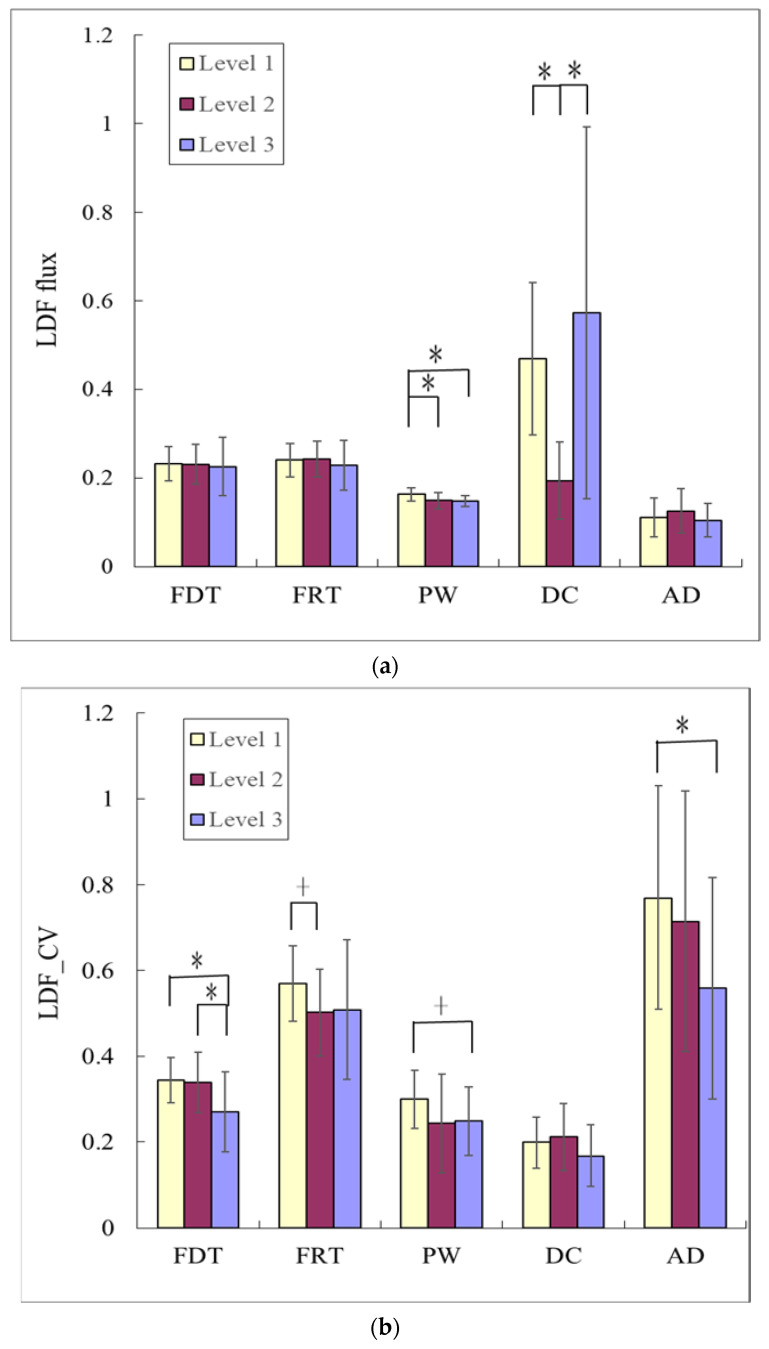
Comparisons of LDF (**a**) flux and (**b**) variability indices among the three groups. Data are mean and SD values. “*” indicates *p* < 0.05; “+” indicates 0.05 < *p* < 0.1. (**a**) For the LDF flux indices, PW was significantly larger in Level 1 than in Level 3 and Level 2, and DC was significantly smaller in Level 2 than in Level 3 and Level 1. (**b**) For the LDF variability indices, FDT_CV was significantly larger in Level 1 and Level 2 than in Level 3, and AD_CV was significantly larger in Level 1 than in Level 3.

**Figure 6:**
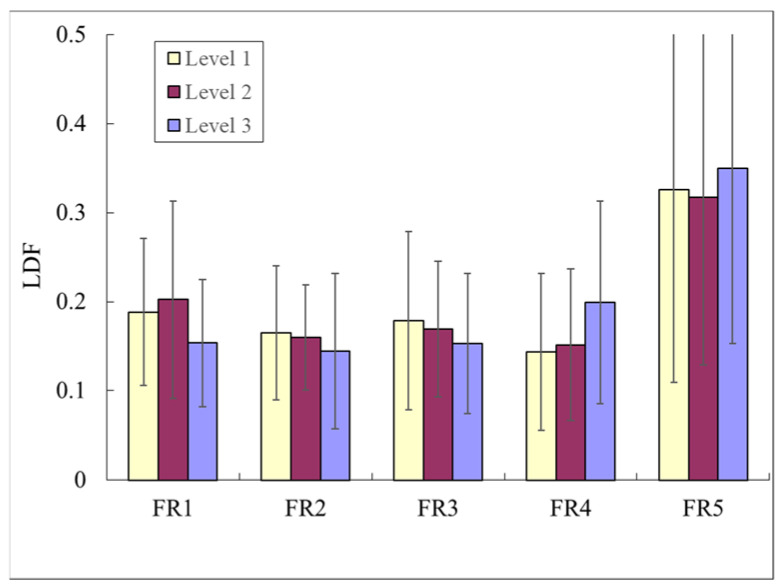
Comparisons of LDF spectral indices among the three groups. Data are mean and SD values. There were no significant differences in all frequency bands among the three groups.

The comparison of the LDF flux indices between the Level-3, Level-2, and Level-1 groups in Figure 5a indicates that PW was significantly larger in Level 1 than in Level 3 and Level 2. DC was significantly smaller in Level 2 than in Level 3 and Level 1. The comparison of the LDF variability indices between the Level-3, Level-2, and Level-1 groups in Figure 5b indicates that FDT_CV was significantly larger in Level 1 and Level 2 than in Level 3. AD_CV was significantly larger in Level 1 than in Level 3. Figure 6 indicates that there were no significant differences in all frequency bands between the three groups. FR4 was smaller (although not significantly) in Level 2 and Level 1 than in Level 3.

**Figure 7:**
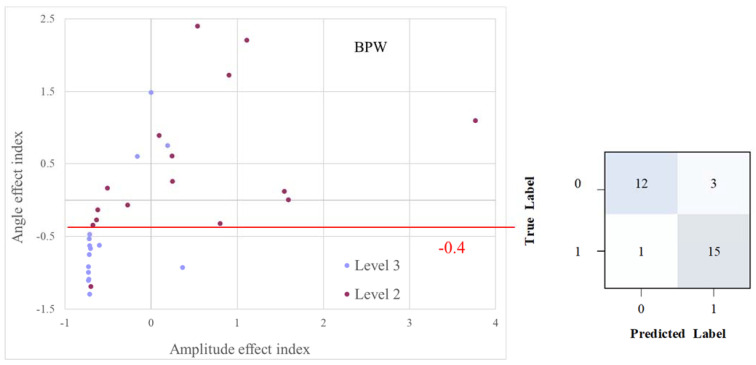
Using relative change distributions of pulse-effect indices to discriminate among groups. For BPW indices, choosing −0.4 as the angle-effect threshold resulted in almost perfect discrimination (AUC = 0.878) between the Level-3 and Level-2 groups.

As illustrated in Figure 7, we can use BPW indices in distribution plots for the relative changes of pulse-effect indices to achieve almost perfect discrimination (AUC = 0.878) between Level 3 and Level 2.

## 4. Discussion

The present findings included significant differences among the pulse waveform and LDF indices of the Level-3, Level-2, and Level-1 groups.

### 4.1. Changes in Pulse Waveform Indices (C_n_ and P_n_)

Regarding cardiovascular effects, competitive sport places strict demands on veteran trail runners [32], and continuously high levels of exercise could have detrimental effects on cardiovascular health [5]. Lighter levels have been noted to be consistently associated with reduced CVD risk, and persistent running over time has been found to be strongly associated with reduced mortality risk [1]. Acute exercise induces a transient increase in aortic stiffness [5], whereas chronic recreational exercise and endurance training can reduce arterial stiffness [3].

Figure 3 and Figure 4 illustrate significant changes in the PPG and BPW pulse indices among the three groups. Pulse waves transmit the propelling force of the heartbeat from the arteries to the peripheral blood vessels, and differences in pulse waveforms could therefore be useful in monitoring changes in vascular properties. *C_n_* represents the spectral distribution of the amplitude of the pulse waveform, and changes in *C_n_* can therefore be related to the propelling condition, such as the propelling force or propagation efficiency.

Exercise may trigger adaptive responses to meet the peripheral blood supply needs at all levels of the cardiovascular system. For example, trained subjects in the Level-2 group were noted to accommodate to the increased cardiovascular demands during exercise better than untrained subjects [3]. Pulse transmission may be affected by changes in arterial stiffness, which in turn alter the distribution of harmonic energy ratios. The changes in *C_n_* were similar in the BPW and PPG waveforms; the most-obvious common change trend was that *C*_2_ was significantly smaller and some of the higher-frequency components (e.g., *C*_4_–*C*_9_) were significantly larger in Level 2 and Level 1 than in Level 3. Lower-frequency components contribute the most to power distribution in arterial pulse waveforms, so they may be more closely related to the pulse transmission condition in the main arteries, and higher-frequency components may be more closely related to the characteristics of peripheral blood vessels. The present findings may therefore demonstrate that changes in *C_n_* are associated with the improved terminal vascular blood perfusion status in Level 1 and Level 2 compared with in Level 3.

Exercise-induced changes in blood vessel stiffness have been proposed to be caused by multiple factors. For example, mechanical fatigue in the elastic elements of the aortic wall has been suggested to be induced by repeated and excessive stress due to reduced HR, increased stroke volume, and aortic distension [3]. Intense exercise-training-induced systemic inflammation might increase arterial stiffness, and the change in sympathetic vascular tone could be another important mechanism [2]. According to the findings for blood vessel stiffness, our methods were found to be useful as an optimized individual cardiovascular risk-stratification tool in addition to the use of traditional risk factors.

As illustrated in Figure 3 and Figure 4, *P*_2_–*P*_6_ of BPW and PPG were both larger in the Level-2 and Level-1 groups than in the Level-3 group, with most of the differences being significant. The phase angle represents the time difference between the arrival time of a certain frequency component and starting point of the pulse waveform; *P_n_* may therefore be related to the stiffness of the peripheral vascular bed [4,9,10]. The long-term effects of exercise may improve this stiffness. Ventricular remodeling and myocardial function might be influenced by the underlying central hemodynamic load in endurance-trained individuals [8]. It is possible that exercise triggers a demand for blood flow from the peripheral tissues. Peripheral blood vessels dilate in response to this demand, which leads to reduced peripheral stiffness and, in turn, a decreased wave velocity of the high-frequency components of the pulse wave. The high-frequency *P_n_* values became smaller, and hence the low-frequency *P_n_* values were relatively increased. This can improve the transmission efficiency of pulse waves and therefore help to improve the efficiency of blood supply to peripheral tissues. The underlying mechanism may be associated with parasympathetic vasodilation, and may contribute to a long-term improvement in blood supply following exercise. These observations further illustrate that the detailed analysis of the pulse waveform frequency can be used to reveal detailed changes in the elastic properties and wave transmission condition of the arterial system.

### 4.2. Changes in Pulse Variability Indices (CV_n_ and P_n__SD)

Changes in indices that described the pulse waveform (*C_n_* and *P_n_*) were similar, whereas changes in the variability indices were the most-prominent differences between the BPW and PPG signals. Regarding BPW variability, several indices were highest in Level 1, with some of the differences being significant compared with Level 3. However, many indices for PPG variability were lowest in Level 1, with the most prominent differences in higher-frequency components: 6–10th harmonic indices presenting a gradual decline in Level 3, Level 2, and Level 1.

Differences in pulse variability indices between BPW and PPG signals may be due to differences in measurement locations (upstream for the radial BPW and downstream for finger PPG) and principles (shallower optical measurement for PPG than for the BPW). The differences in BPW variability could be more closely related to the physiological regulation in the main arteries, which implies a consistent effect of exercise on the improvement of peripheral circulatory blood supply (e.g., the propelling force and transmission speed) in the upstream and downstream measurements. Changes in the BPW variability indices may also be associated with parasympathetic activity. The large difference in BPW variability indices between the Level-2 and Level-1 groups may be associated with parasympathetic activity predominance, which causes blood vessels to dilate and hence increase the blood supply. The differences in PPG variability indices could be more closely related to the stability of the peripheral blood supply. The signal is more obvious when the peripheral blood circulation is sufficient, which increases the signal-to-noise ratio; the variability value was therefore smaller in the Level-2 and Level-1 groups. Since the terminal tissues of the body are far from the heart, and the structure of the terminal vascular bed and blood supply mechanism are more complicated, the present results illustrate that regulatory mechanisms can differ between the main arteries and terminal blood vessels; hence there were obvious differences between PPG and BP signals in the variability indices. The present results also suggest that measuring pulse variability indices at different sites (e.g., radial BPW and finger PPG) can be helpful for assessing the regulatory abilities at different levels of the vascular system following exercise.

Skeletal muscle characteristics might influence BP in Level 3 and peripheral blood-flow resistance during exercise [2]. The increased endothelium-dependent dilation might be attributable to mechanical compression of vessel walls during exercise, increasing their resistance, followed by blood flow release after exercise cessation, which induces a sharp increase in shear stress in the vessel walls [4]. Stronger vasomotor reaction has been noted in microvessels [32], which could be associated with the increased BPW variability indices.

HRV is another widely used index category for evaluating cardiovascular regulation activity. It is also used as an indicator of the recovery state in individuals in Level 2 [33], whose HRV profile differed from that in sedentary Level 3 subjects, with overall increases in HRV and parasympathetic cardiac modulation [34]. Increases were noted in the time-domain HRV parameters that were suggested to be related to positive adaptation from endurance-running training in Level-2 individuals [6,33]. This was similar to the change observed in most BPW variability indices, which was that the Level-3 group had smaller values than the Level-2 and Level-1 groups. However, this trend did not occur in PPG, which implies that this mechanism affected the main arteries but not the peripheral blood vessels. Furthermore, elevated HRV values remained in Level 1 compared with Level 2.

Autonomic imbalance has been suggested to explain the changes in frequency-domain HRV indices [34]. Exercise training reduces sympathetic burst activity in the muscles. Training for sport competitions promotes a long-term increase in parasympathetic tone with concomitant inhibition of sympathetic tone in the resting state [6,7,34,35]. For example, the higher-frequency spectral power, an index of parasympathetic activity, has been noted to be significantly higher in the Level-2 group than in the other groups [36].

The changes in BPW pulse variability indices that we observed were similar to those in HRV indices. The possible parasympathetic predominance could contribute to the long-term increase in vasodilation and improvement in blood supply in Level 2 and Level 1. This suggests that cardiovascular variability analysis can be expanded to explain the changes in the physiological regulatory activity in the main artery and peripheral blood vessels following exercise.

### 4.3. LDF Indices

Comparing LDF flux indices revealed differences among the Level-3, Level-2, and Level-1 groups. Figure 5a illustrates that DC was significantly larger in Level 1 than in Level 2, and that PW was significantly larger in Level 1 than in Level 3 and Level 2. DC (the mean blood flux) is related to the blood flow while PW (the pulse width) is related to the perfusion efficiency of blood flow driven by pulse BP through arteriolar openings into tissues. These results therefore illustrate that the Level-1 group may have more perfusion or greater efficiency in the resting state.

The comparison of the LDF variability indices in Figure 5b indicates that several of them were larger in Level 1: FDT_CV was significantly larger in Level 1 and Level 2 than in Level 3, and AD_CV was significantly larger in Level 1 than in Level 3. Considering FDT_CV as an example, FDT is defined as the time delay between the R peak on ECG and the LDF foot point of each pulse, and it is related to the change in the speed of pulse wave transmission when arterial stiffness changes. Previous studies have found that exercise can induce a decrease in PWV [7,9]. There were no significant differences in FDT in the present study, whereas FDT_CV was significantly larger in Level 2 and Level 1. This may represent a change in vascular elasticity that, although not obvious, did affect vascular elastic regulation. A larger FDT_CV may be associated with parasympathetic predominance, thus contributing to improved blood supply in Level 2 and Level 1.

Regarding the LDF spectral indices, no significant differences were noted among the three groups (Figure 6). It is worth noting that the RECs of FR4 were smaller (although not significantly) in both Level 2 and Level 1 than in Level 3. Since changes in the FR4 frequency band may be related to changes in baroreflex activity [23], parasympathetic activity may predominate in the exercise groups, resulting in a decrease in the REC of FR4, which in turn is related to the improved peripheral blood supply.

### 4.4. Distinguishing Level 1 from Level 2

Based on the above conjectures, the present findings illustrate that comparing pulse parameters between the Level-2 and Level-3 groups can be used to identify and evaluate the physiological benefits of exercise. For waveform indices (*C_n_* and *P_n_*), several were larger in Level 2 and Level 1, and the common physiological meaning may be that pulse wave transmission is more efficient, which helps to improve blood supply perfusion.

Regarding pulse variability indices (*CV_n_* and *P_n_*_*SD*), some BPW variability indices were larger in Level 2 and Level 1, which may be attributed to parasympathetic predominance in the arterial transmission of pulse waves, which may further contribute to the improvement of the peripheral blood flow. Some PPG variability indices were smaller in Level 1. This suggests morestable peripheral blood flow, reflecting a moreadequate blood flow in Level 1.

Moreover, several LDF indices can be used to monitor the improved blood-flow perfusion condition in the exercise-trained and Level-1 groups. For example, DC is helpful for observing the blood flow, and PW reflects the efficiency of blood flow perfusion. It was also found that the blood flow variability indices were larger in Level 2 and Level 1, which may indicate parasympathetic activity predominance and is related to the improvement of blood flow caused by peripheral vasodilation.

The results of this study have indicated that several pulse wave and blood flow indices can be used to effectively distinguish between Level-1 and Level-2 individuals. These indices include some smaller PPG variability indices, some larger BPW variability indices, and some LDF indices such as larger PW, DC, and AD_CV. As mentioned above, the physiological significance of these indices could be related to the parasympathetic predominance and improved blood-flow condition in Level 1, which can therefore be applied to the screening of outstanding Level 2s.

### 4.5. Classification by Analysis of Pulse-EffectIndices

As noted in Figure 7, the relative changes in the amplitude and angle effect indices can be used to achieve almost perfect discrimination between Level 2 and Level 3 (BPW; AUC = 0.878) by setting the appropriate filter criteria. These changes can be used to assess physiological advantages induced by exercise. This finding was derived from the comparison of pulse indices as illustrated in Figure 3 and Figure 4, which enable the statistical characteristics of the indices to be understood more completely. This approach can effectively compensate for the uncertainty of the effect on the classification ability due to the black-box property in the underlying physiological mechanism of artificial intelligence analysis. Another advantage of the analysis of pulse-effectindices is that the accumulation of a very large amount of data—as needed for statistical approaches such as in artificial intelligence analysis—is not required. The algorithm can effectively classify Level 2 and Level 3 when using a slightly smaller sample, which is useful for early-stage research in classifying different blood supply effects by helping to establish the initial exploration direction. As the number of subjects increases, the algorithm will also assist in the subsequent selection of features for artificial intelligence analysis.

Selected studies are presented in Table 2 for their user friendliness, accuracy, required time, noninvasive evaluation, and cost, which can be divided into biochemical, subjective, cognitive and physiological variables. The sensors used to measure the physiological responses were analyzed and have been reported for their sampling frequency, sensors’ accuracy, and location [37]. EMG with a wireless transmission module has been validated in the lab setting while the user friendliness was not ideal for long-term continuous monitoring of physiological activities [38]. The Borg Rating of Perceived Exertion (RPE) has been reported as a safety measure [39]; however, it was reported that Borg RPE demonstrated lower sensitivity [40].

## 5. Conclusions

The present findings are of importance for exercise science based on the implications that the cardiovascular impact of endurance running is dependent on levels of running performance. The following four conclusions can be drawn from the results of this study:

1. Significant differences were noted in pulse and LDF indices between groups. These could be used to evaluate advantageous cardiovascular effects provided by exercise.
2. The present method of pulse waveform analysis can be used to discriminate Level 1 from Level 2, and hence aid in the screening of outstanding Level 2s.
3. Using relative changes in the amplitude and angle-effect indices can achieve almost perfect discrimination between Level 2 and Level 3 (AUC = 0.878 for BPW). This could be useful for early-stage research in classifying the different effects on blood supply, and can also assist in feature selection in subsequent artificial intelligence analysis.
4. There is a need for convenient and immediate evaluation tools for evaluating the benefits of exercise on the cardiovascular system. The present findings may contribute to the development of technologies and devices for evaluating exercise-induced physiological effects.

The present study was limited by the relatively small sample because it is more difficult to recruit professional runners as subjects. Future studies should focus on accumulating more data to reinforce the reliability of discrimination analysis. For example, age may cause interference effects; more data need to be accumulated to clarify their possible effects on the present indices. Furthermore, collecting more data will also facilitate future AI analysis to construct reliable classification models.

## Figures and Tables

**Table 1:** Characteristics of the study subjects.

Group	Level 3	Level 2	Level 1
Subject number	15	16	13
Age	29.5 ± 13.4	25.9 ± 3.1	33.2 ± 5.9
HR	75.93 ± 14.90	58.3 ± 10.00	55.29 ± 8.25
HR_CV	0.061 ± 0.016	0.067 ± 0.014	0.061 ± 0.017
BMI	23.39 ± 1.71	23.33 ± 1.80	22.20 ± 1.70

**Table 2:** Comparison of different methods for evaluating physiological effects of running. “O”: good performance; “∆”: acceptable performance.

	Pulse and Blood Flow Waveform Analysis	Electromyography(EMG)	Blood Test	Rating of Perceived Exertion (RPE)
User friendliness	O	∆		O
Accuracy	∆	O	O	∆
Required time	O		∆	O
Noninvasive evaluation	O	∆		O
Cost	O	∆	O	O

## Data Availability

The datasets generated during and/or analysed during the current study are available from the corresponding author on reasonable request.

## Supplementary Materials

The following supporting information can be downloaded at: https://zenodo.org/record/7710453#.ZAle9hVBy5c.
